# Helping cancer patients quit smoking using brief advice based on risk communication: A randomized controlled trial

**DOI:** 10.1038/s41598-018-21207-1

**Published:** 2018-02-09

**Authors:** William H. C. Li, M. P. Wang, K. Y. Ho, Katherine K. W. Lam, Derek Y. T. Cheung, Yannes T. Y. Cheung, T. H. LAM, Sophia S. C. CHAN

**Affiliations:** 1 School of Nursing, The University of Hong Kong, HKSAR, Hong Kong, China; 2 School of Public Health, The University of Hong Kong, HKSAR, Hong Kong, China

## Abstract

This randomized controlled trial aimed to examine the effectiveness of a smoking cessation intervention using a risk communication approach. A total of 528 smoking cancer patients were randomly allocated either into an intervention group (n = 268) to receive brief advice based on risk communication by a nurse counselor or a control group (n = 260) to receive standard care. Subjects in both groups received a smoking cessation booklet. Patient follow-ups were at 1 week and at 1, 3, 6, 9 and 12 months. No significant differences were found in self-reported point-prevalence 7-day abstinence between the intervention and control groups at 6 months (15.7% vs 16.5%; OR 0.94, 95% CI 0.59–1.50). The rate of at least 50% self-reported reduction of smoking at 6 months, was higher in the intervention group than in the control group (16.8% vs 12.3%; OR 1.43, 95% CI 0.88–2.35). The biochemically validated quit rate at the 6-month follow-up was higher in the intervention group than in the control group (5.2% vs 3.8%; OR 1.38, 95% CI 0.60–3.16). These data suggest that advice based on risk communication was not effective for quitting but improved the rate of smoking reduction among smoking cancer patients.

## Introduction

Tobacco smoking causes many types of cancer, including cancer of the lung, larynx, oesophagus, stomach, liver, pancreas, kidney, ureter, bladder and colo-rectum, as well as acute myeloid leukaemia^1^. Although recent advances in cancer treatment have dramatically improved the survival rate, cancer patients who continue smoking bear extra risks in terms of all-cause mortality as well as reduced treatment effectiveness and survival time^2^. Smoking could also lower the efficacy of cancer treatment^3,4^ and add to the risk of post-treatment side effects^5^. On the contrary, there is evidence that cancer patients who quit smoking could reduce the risk of disease advancement^6^, improve prognosis, minimize adverse treatment-related effects and promote quality of life^7^. Given the detrimental effects of smoking and the beneficial effects of cigarette smoking cessation, it is essential for healthcare professionals to help cancer patients quit smoking.

Smokers who had been diagnosed with cancer attended to out-patient clinics present an excellent ‘teachable moment’ for smoking cessation interventions, as it provides a valuable opportunity for them to stop smoking cessation to improve their health^8,9^. Healthcare professionals can also take this golden opportunity to promote smoking cessation while they are waiting for medical consultation. However, cigarette smoking is addictive and quitting is very difficult, with a high rate of relapse, particularly among those chronic patients with high nicotine dependency^10,11^. Previous studies showed that about one third of cancer patients in Western countries and almost 50% smokers with lung cancer in USA continued to smoke after receiving a cancer diagnosis^12–15^. Our recent study found that approximately 14% of patients continued smoking after receiving a cancer diagnosis^16^. Many cancer patients who continued smoking had misconceptions that a moderate amount, such as half a pack of cigarettes per day, might not be detrimental to their health^16^. Some smokers said that it was too late to stop smoking as their cancer had reached a later stage^16^. Therefore, it is vital for healthcare professionals to design smoking cessation interventions that use strong warning to clearly communicate the risk of continued smoking to this group as a strategy to enhance their motivation to quit.

## Theoretical framework

According the Theory of Planned Behaviors, smokers with positive beliefs in attitudes and subjective norms that contribute to stronger intention in quitting will be more likely to quit smoking^17^. Our intervention therefore aimed to change the patients’ attitudes and their subjective norms through risk communication, which was used in a previous study, showing a 24% self-reported quit rate at 6 months in lung cancer patients^18^. To guide the risk communication, a core construct of the Transtheoretical Model, named decisional balance that emphasizes the importance of balancing the pros and cons of a behavioral change^19^, was applied. Individuals who consider that the cons outweighs the benefits, are more like to continue smoking. Whereas those who perceive greater benefits from quitting smoking are more motivated to change. Accordingly, during the intervention, patients were informed the advantages of quitting smoking and drawbacks of not quitting with regard to cancer.

Behavioural intervention has been shown to be helpful in improving cessation rates in smoking cancer patients in several randomized controlled trials (RCTs). In head and neck cancer patients, significant differences were shown in a behavioural intervention group compared with a usual care group (47% vs. 31%; n = 184, *p* = 0.05) at 6-month follow-up^20^. Another RCT showed that, compared with those given only standard care, hospitalized cancer patients given behavioural intervention had a higher quit rates (21% vs. 14%, n = 28)^21^. Wakefield *et al*. applied behavioural intervention in an RCT to help patients with mixed cancer sites and demonstrated enhanced smoking cessation (29% vs. 18%; n = 137, *p* = 0.32)^22^. However, these RCTs were limited by small sample size and no RCTs have been conducted on Chinese cancer patients who smoke in Hong Kong and elsewhere.

The present RCT with a large sample size aimed to study the effectiveness of a face-to-face individualized brief risk communication to encourage patients with cancer to stop smoking. We hypothesized that participants in the intervention group would (i) have a higher smoking cessation rates by self-reporting and biochemical validation and (ii) have a higher self-reported rate of having reduced daily cigarette consumption by at least 50%.

## Methods

### Ethical Review

This study was approved by the Institutional Review Board of the University of Hong Kong and the Hospital Authority of Hong Kong West Cluster (Reference: UW 12-113) and was registered on the Clinical Controlled Trials registry as NCT01685723. Eligible subjects were invited to participate in the study after being informed of its purpose. They were informed that their participation was voluntary and without prejudice, and given the opportunity to refuse to participate. Written informed consent was obtained from all eligible subjects prior to randomization and all the research procedures were in compliance with the Helsinki.

### Study Participants

Smokers who attended medical follow-up visits at outpatient clinics of the Clinical Oncology Departments of five major hospitals in different regions of Hong Kong and who met the inclusion criteria were invited to participate. The inclusion criteria were subjects who (a) had smoked at least weekly in the past 6 months; (b) had been diagnosed with cancer not limited to smoking-induced cancers; (c) were in any of the cancer stages 0, I, II, III, or IV; and (d) aged 18 above and able to communicate in Cantonese. Subjects were excluded if they had already participated in other smoking cessation programs, had unstable medical conditions, or had a poor cognitive state or mental illness as advised by the doctor in charge and noted on their medical records.

### Sample Size

In a previous study which examined the effectiveness of an intervention based on risk communication approach in motivating 137 smoking patients with mixed cancer sites to quit, the self-reported 7-day point prevalence quit rate at the 6-month follow-up was 29% for the intervention group and 18% for the control group^22^. To detect a statistical difference at the two-sided 5% significance level and a power of 80%, 233 subjects were required for each group. Based on the 15% subject attrition rate at the 6-month follow-up that was experienced in a local RCT on smoking cessation intervention for cardiac patients^23^, 548 subjects were required in total.

### Interventions

The intervention proposed is a behavioural intervention as it was repeatedly delivered in order to initiate behavioural change^24^. In particular, subjects received face-to-face individualized brief advice based on risk communication for 15–30 minutes from the smoking cessation counselors, followed by exhaled carbon monoxide level assessment measured by using smokerlyzer (Bedfont® Scientific, Harrietsham, UK). Counselors delivered brief advice based on a specifically-designed risk communication leaflet that warns about the risks of continued smoking for subjects’ cancer treatment and prognosis^7^. A high intensity counseling session (>30 minutes) may be able to boost the quit rate^25^, but is not practicable or feasible in busy clinical settings. In addition, subjects may lose patience while they are waiting for medical consultation in out-patient clinics. Thus the intervention was kept short (within 15–30 minutes). The risk communication component focused on the relationships between smoking and cancer diagnosis, treatment and prognosis as a trigger to think about quitting. Subjects in the experimental group also received a booster intervention via telephone during follow-up at 1 week. The booster intervention aimed to assess the progress of and barriers to the subjects’ action plans and identifying individual difficulties and facilitators towards quitting. To support the subjects to quit smoking, the counselors would provide information on how to handle withdrawal symptoms. However, nicotine replacement therapy were not provided in this study as it may not be appropriate for patients with some diseases, particularly those undergoing medical treatment^23^. In addition, the acceptance of and adherence to such therapy is low even among Chinese smokers who want to quit^26^.

Subjects in the control group received only standard care without risk communication, but had the same follow-up sections as the intervention group to receive diseases support. Subjects in both groups received a generic standard self-help smoking cessation booklet.

### Study Design

This was a single-blinded simple individual RCT. Smoking cessation counselors approached the patients at the oncology outpatient clinic and queried their smoking and cancer diagnosis status. A list of computer-based random numbers was generated by SPSS software for each hospital to allocate consented subjects into groups. Counselors opened a serially-numbered sealed-opaque envelope to ensure each subject’s allocation was concealed after baseline survey. Such a masking procedure avoids bias in completing the baseline questionnaire and the design follows the recommendations of CONSORT’s Statements to guide randomized controlled studies (http://www.consort-statement.org/).

### Study Assessments and Outcomes

The primary outcome was self-reported 7-day point-prevalence smoking abstinence at 6-month follow-up. The secondary outcomes include self-reported 7-day point-prevalence smoking abstinence at 12-month follow-up, biochemically validated quit rate at 6-month follow-up and percentage of patients reduced smoking by at least 50% at 6- and 12-month follow-up compared to baseline.

### Demographics and Smoking Characteristics

Baseline data included smoking history, demographic, socioeconomic and clinical characteristics obtained from each subject using a structured questionnaire administered by a trained nurse counselor. The content of the structured questionnaire included smoking-related information, such as daily cigarette consumption, nicotine dependency as assessed by the Fagerstrom test^27^, the stage of readiness to quit smoking^19^ and previous quit attempts.

### Data Collection

The data collection period including the subject recruitment and follow-up were between September 2012 and March 2015. For both groups, all the consecutive follow-ups at 1 week and 1, 3, 6, 9 and 12 months were conducted by trained nurse counselors via telephone. A structured questionnaire was adopted to assess subjects’ smoking and quitting history, risk perceptions of smoking^28^, illness perception^29^, intention to quit smoking (stage of readiness to quit)^10^ and self-efficacy to quit smoking^30^.

Subjects who quit smoking successfully at 6 months were invited to take the biochemical validation test, which comprised measurement of cotinine in saliva (<115 ng/mL NicAlert strips (www.nymox.com)^31^ and an exhaled carbon monoxide test (<4 ppm to confirm quitting or <9 ppm for smoking reduction)^32^. Subjects were reimbursed for travel to the biochemical validation test and lucky draws (cash incentives) were offered to boost the response rates for the follow-ups. Ten subjects from the intervention group who had not quit were invited for a recorded 10- to 20-minute individual process evaluation in the form of face-to-face interviews by research assistants at 12-month follow-up. They were asked two open-ended questions such as “What do you think of the intervention leaflet? Was it useful or not?” and “Did you reduce your smoking after joining our program?” The responses were recorded and translated verbatim for analysis.

### Statistical Analysis

Quantitative data were analysed using the Statistical Package for Social Science (SPSS Inc., Chicago, IL, USA) version 23.0 for Windows. Baseline demographic characteristics of subjects between intervention and control group were compared using Chi-square test or Fisher’s exact test for categorical variables and t-test for continuous variables. The effect on smoking cessation at 6 and 12 months was compared by unadjusted odds ratios (ORs) using logistic regression. Predictors of abstinence at 6 and 12 months were analysed by multivariable logistic regression to yield the adjusted ORs. Intention-to-treat analysis^33^ was used in this study with all subjects (N = 528) were included in the analysis and all non-responses were treated as non-quitters. The significance level of all analyses was set at 5%. Qualitative data from the process evaluation were tape-recorded and transcribed verbatim. Data were summarized to evaluate the effect of the intervention as perceived by the participating subjects.

## Results

### Baseline Study

**Figure 1:**
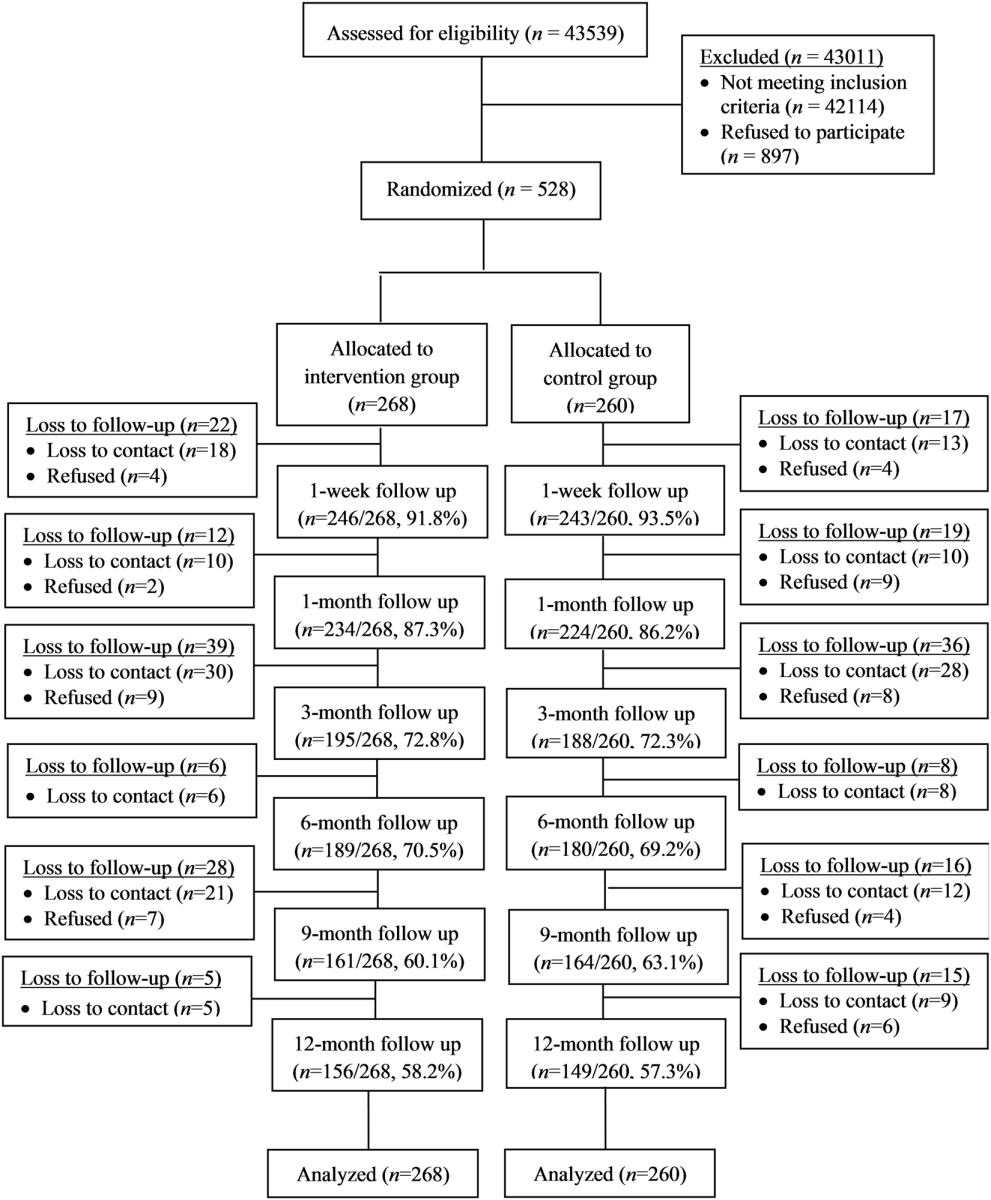
The Consolidation of Standards for Reporting Trials (CONSORT) Flowchart.

Of the 43,539 subjects screened at the oncology units during the study period, 1425 (3.3%) subjects were eligible. However, 897 patients either showed no interest in joining the study or were unavailable for the upcoming interventions. The remaining 528 patients were randomly assigned to the intervention group (n = 268) and control group (n = 260). The retention rates for the intervention and control groups were 189/268 (70.5%) and 180/260 (69.2%) at 6 months, respectively. The retention rates were 156/268(58.2%) and 149/260 (57.3%) for intervention and control groups, respectively at the 12-month follow-up. The principle of intention-to-treat was applied and the sample size included in the final analysis was 528. The Consolidation of Standards for Reporting Trials (CONSORT) flowchart in Fig. 1 indicated the process of the study and no adverse effects were reported throughout the trial.

In Table 1, the average age of the 528 subjects was 58.9 ± 12.3 years. About one-third of the subjects were employed (n = 201, 38.1%) while 38.9% of them had retired (n = 205). Most were pre-contemplating quitting smoking (72.0%, n = 380) and smoked 12.5 ± 8.0 cigarettes daily. They smoked for 42.0 ± 13.2 years on average, with mild to moderate nicotine dependence (scored 3.1 ± 2.2). Over 80% of the cancers had been first diagnosed at the beginning of the program in both the intervention and control group subjects and the distributions of cancer locations among the groups were similar. About 40% of the subjects from both groups perceived their health status as fair. Except for the distribution in education attainment (*p* = 0.02), no statistically significant differences were found between the intervention and control groups in the demographic characteristics at baseline.

**Table 1:** Baseline characteristics of the subjects.

	Intervention^b^ (n = 268)	Control^c^ (n = 260)	*P-value*
Gender, n (%)			
Male	234 (87.3)	221 (85.0)	0.44
Female	34 (12.7)	39 (15.0)	
Age, mean ± SD	59 ± 12.8	59 ± 11.7	0.70
Site of primary cancer^a^, n (%)			0.19
Lung	34 (12.7)	23 (8.8)	
Colorectal	51 (19.0)	55 (21.2)	
Prostate, Testicle	22 (8.2)	17 (6.5)	
Liver, Bile duct	28 (10.4)	31 (11.9)	
Stomach, Pancreas, Small intestine	19 (7.1)	12 (4.6)	
Kidney, Bladder	6 (2.2)	7 (2.7)	
Nasopharynx	46 (17.2)	40 (15.4)	
Oral, tongue, Tonsil, Vocal cord	6 (2.2)	8 (3.1)	
Throat, Esophagus, Thyroid	29 (10.8)	24 (9.2)	
Breast	4 (1.5)	17 (6.5)	
Bone, Big cell, Sarcoma, Skin, thigh	5 (1.9)	6 (2.3)	
Lymphoma	10 (3.7)	9 (3.5)	
Cervical, Ovary	2 (0.7)	6 (2.3)	
Nervous system, Neck, Brain	5 (1.9)	2 (0.8)	
Missing	1 (0.4)	3 (1.2)	
Educational attainment^a^, n (%)			0.02*
Primary school or below	115 (43.2)	85 (32.7)	
Secondary	137 (51.5)	163 (62.7)	
Tertiary or above	14 (5.3)	9 (3.5)	
Missing	2 (0.7)	3 (1.2)	
Marital status, n (%)			
Single	23 (8.6)	24 (9.2)	0.32
Married/cohabitation	205 (76.5)	202 (77.7)	
Divorced/separated	34 (12.7)	23 (8.8)	
Widowed	6 (2.2)	11 (4.2)	
Employment status^a^, n (%)			
Retired	100 (37.3)	105 (40.4)	0.78
Unemployed	60 (22.4)	59 (22.7)	
Employed	105 (39.2)	96 (36.9)	
Missing	3 (1.1)	0 (0)	
Stages of cancer (exclude missing)^a^, n (%)			
Stage 0, I	36 (13.4)	30 (11.5)	0.58
Stage II	48 (17.9)	39 (15.0)	
Stage III	39 (14.6)	37 (14.2)	
Stage IV	28 (10.4)	37 (14.2)	
Not identified	112 (41.8)	109 (41.9)	
Missing	5 (1.9)	8 (3.1)	
Diagnosis Status^a^, n (%)			
First diagnosis	212 (79.1)	215 (82.7)	0.35
Recurrence	24 (9.0)	18 (6.9)	
Missing	32 (11.9)	27 (10.4)	
Previous serious quit attempts for 24 hours^a^, n (%)			0.94
Yes	185 (69.0)	178 (68.5)	
No	83 (31.0)	81 (31.2)	
Missing	0 (0)	1 (0.4)	
Stage of readiness to quit, n (%)^a^			0.41
Pre-contemplation stage	194 (72.4)	186 (71.5)	
Contemplation stage	33 (12.3)	30 (11.5)	
Preparation stage	33 (12.3)	25 (9.6)	
Action stage	7 (2.6)	13 (5.0)	
Missing	1(0.4)	6 (2.3)	
Years of regular smoking^a^, mean ± SD	42 ± 14.1	42 ± 12.3	0.59
No. of cigarette consumed per day (baseline), mean ± SD	13 ± 7.8	12 ± 8.1	0.55
Fagerstrom Nicotine Dependence Score^a^, mean ± SD	3.2 ± 2.2	3.0 ± 2.2	0.39

^a^Missing data are excluded.

^b^Brief advice based on risk communication.

^c^Usual care.

### Intervention outcomes

The outcomes for effectiveness of the smoking cessation intervention using a risk communication approach are shown in Table 2. No statistically significant differences were found in the 7-day point-prevalence of smoking abstinence or in self-reported quit attempts at 6 months and 12 months. However, the biochemically validated quit rate was higher in the intervention group than in the control group (5.2% vs. 3.8% at 6 months and 5.6% vs. 4.6% at 12 months) although significance was not reached. Self-reported smoking reduction of at least 50% at 6 and 12 months was also higher in the intervention group than in the control group (16.8% vs. 12.3%; p = 0.14 at 6 months and 10.4% vs. 9.6%; p = 0.75 at 12 months). There were a total of 47 subjects who had passed away as of the 12-month follow up and there was no significant difference in the mortality rate between the intervention and control groups.

**Table 2:** Quit rate, smoking reduction rates and quit attempts in intervention and control groups

	Intervention group^c^ N = 268	Control group^d^ N = 260	*P-value*
Primary outcome at 6 months^a^			
Self-reported 7-day quit rate	42 (15.7)	43 (16.5)	0.79
Secondary outcome^a^			
Self-reported 7-day quit rate			
1 month	43 (16.0)	54 (20.8)	0.16
3 months	49 (18.3)	45 (17.3)	0.77
9 months	40 (14.9)	49 (18.8)	0.23
12 months	40 (14.9)	53 (20.4)	0.10
Biochemically validated quit rate			
6 months	14 (5.2)	10 (3.8)	0.45
Change of stage of readiness between 1- and 6-month			
Decreased	26 (9.7)	18 (6.92)	0.37
No Change	161 (60.1)	167 (64.2)	
Increased	44 (16.4)	37 (14.2)	
Self-reported reduction in daily cigarette consumption ≥50%^b^			
6 months	45 (16.8)	32 (12.3)	0.14
12 months	28 (10.4)	25 (9.6)	0.75
At action stage of readiness to quit			
6 months	42 (15.7)	44 (16.9)	0.67
12 months	37 (13.8)	49 (18.8)	0.12
Quit attempt to abstain smoking for >24 hrs since last assessment^b^			
6 months	21 (7.8)	21 (8.1)	1.00
12 months	20 (7.5)	24 (9.2)	0.35

^a^By intention-to-treat analysis: assume all non-responded follow-up patients as current smokers, who did not make a quit attempt over the period and did not change their behavior compared to baseline.

^b^Quitters excluded from numerator.

^c^Brief advice based on risk communication.

^d^Usual care.

### Prediction factors in follow-up periods

In Table 3, logistic regression results indicated that the rates of quitting smoking in the intervention group at 6 months were similar in both crude (OR, 0.94; 95% CI, 0.59–1.50) and adjusted (AOR, 1.03; 95% CI, 0.63–1.70) models. Similar results were also found in 12 month follow up with crude ratio (OR, 0.69; 95% CI, 0.44–1.08) and adjusted models (AOR, 0.75; 95% CI, 0.47–1.20). After adjusting for sociodemographic variables at baseline, the results showed that gender did not predict the likelihood of quitting smoking at 6 months (OR, 0.85; 95% CI, 0.42–1.73) and 12 months (OR, 1.03; 95% CI, 0.51–2.08).

**Table 3:** Factors predicting smoking cessation at 6 and 12 months follow-up.

Variables^a^	6-month	*P-value*	12-month	*P-value*
Unadjusted model	n = 528		n = 528	
Intervention group^b^	0.94 (0.59–1.50)	0.79	0.69 (0.44–1.08)	0.10
Control group^c^	1.00		1.00	
Adjusted model^d^	n = 515		n = 515	
Study group				
Intervention group^b^	1.03 (0.63–1.70)	0.90	0.75 (0.47–1.20)	0.22
Control group^c^	1.00		1.00	
Sex				
Male	0.85 (0.42–1.73)	0.66	1.03 (0.51–2.08)	0.94
Female	1.00		1.00	
Age	1.01 (0.99–1.03)	0.32	1.01 (0.99–1.04)	0.22
Education				
Primary or below	0.91 (0.27–3.10)	0.88	1.00 (0.30–3.30)	1.00
Secondary	1.28 (0.40–4.17)	0.68	1.11 (0.35–3.54)	0.85
Post-secondary	1.00		1.00	
Baseline daily cigarette consumption	1.02 (0.99–1.04)	0.30	0.97 (0.94–1.01)	0.09
Baseline past quitting attempt				
Yes	0.79 (0.46–1.34)	0.38	0.73 (0.45–1.19)	0.21
No	1.00		1.00	

^a^By intention-to-treat analysis: assume all non-responded follow-up patients as current smokers and they did not made a quit attempt over the period and did not change their behaviour compared to baseline.

^b^Brief advice based on risk communication.

^c^Usual care.

^d^Model adjusted for all the variables listed.

Similar null findings were observed for other predictors such as age, education attainment, baseline daily cigarette consumption and previous quit attempts.

### Process evaluation

There were a total of 10 non-quitters who consented and were interviewed from the intervention group after the trial. Some of them reported that they reduced their smoking because of physical symptoms such as suffering from shortness of breath after physical activity. Many thought that because they already suffered from cancer so they would like to smoke to relax or ease the side-symptoms of the treatment. Some of them mentioned that even non-smokers ultimately die and that therefore there was nothing to be afraid of or worried about with regard to smoking. Many of them said that they did read the specifically designed risk communication leaflet with regard to cancer and smoking but that they usually put it away afterwards and that it was easy to lose.

## Discussion

The present study examined the effectiveness of a brief intervention based on risk communication to help Hong Kong Chinese cancer patients quit smoking. To the best of our knowledge, this is the first and largest RCT conducted to evaluate a smoking cessation intervention to help cancer patients quit smoking in Chinese population. One strength of the study is that power analysis was used to estimate the sample size, which reduced the chance of committing type II error. In addition, a large sample of subjects were recruited from outpatient clinics of five major hospitals in different regions of Hong Kong, which enhance the generalizability of the findings.

The results showed that biochemically validated quit rate at the 6-month follow-up was higher in the intervention group than in the control group though it was not statistically significant. However, there was no significant difference in the primary outcome, 6-month self-reported 7-day abstinence, between the intervention and control groups. About three quarters of the subjects were not prepared to quit and some of them even ignored the smoking cessation advice upon recruitment. There are some possible reasons to explain the non-significant findings. First, despite Hong Kong is a westernized city; many people are still subject to the influence of Confucianism, especially its associated notion of fatalism^16^. Some cancer patients might believe that nothing could be done to change their fate and hence decided not to quit smoking even after they were told that continued smoking would be further detrimental to their health. Second, our previous study showed that most subjects quitted smoking following cancer diagnosis^16^. In general, subjects in the present study were not in the early diagnosis stage but already had 5 years in average since the diagnosis of cancer. About 72.9% subjects were in the pre-contemplation stage of quitting smoking and most of them were reluctant to quit smoking. If most of the smokers who wanted to quit had quit before being recruited in our RCT, the subjects included probably represent the most hard-core smokers. Our intervention could be too brief and inadequate to make an impact on such smokers. The results are also in-line with some previous reviews indicating that comprehensive intervention might be more effective than brief advice to promote smoking cessation^34–37^. However, providing comprehensive smoking cessation to cancer patients is not feasible in Hong Kong busy clinical settings, as in general, it takes more than 30 minutes to implement. One alternative strategy to enhance the brief intervention effect is to refer and motivate smokers to utilize the existing smoking cessation services in Hong Kong, in particular for everyone who needs more counseling. We recently conduct a RCT to test the effectiveness of a brief smoking cessation intervention combines different components, i.e. brief advice based on the AWARD (ask; warn; advise; refer; and do it again) model, referral and follow-ups, among people attending emergency departments. The preliminary results indicated that such approach is effective in promoting smoking cessation among people attending emergency departments (7-day point prevalence quit rate - Intervention group: 10.4% vs Control group: 6.6%).

Results indicated that self-reported smoking reduction of at least 50% at 6 and 12 months was higher in the intervention group than in the control group. The results also revealed that some cancer patients who were reluctant to quit were willing to reduce the number of cigarettes smoked per day. Therefore, another potential option would be to help cancer patients who smoke to reduce the number of cigarettes smoked gradually, with the ultimate goal of complete cessation. A meta-analysis summarizing 10 studies on quitting at once verse gradually quitting indicated that patients should be allowed to select their own schedules of quitting, such as to quit immediately or to reduce the number of cigarettes smoked progressively^38^. It is anticipated that patients who have an opportunity to decide on their own treatment may feel more eager to comply with instructions as a result of an increase in autonomy.

Misconceptions about smoking was shown in the risk communication leaflet and such information was repeated during telephone counseling. However, subjects in interviews mentioned that the leaflet was easy to lose and that its message was not impressive enough. Thus, a digital reminder via a smartphone application might be explored as an alternative electronic approach. Many studies have already evaluated the effectiveness of using such digital reminders as a form of cessation intervention with positive feedback^39–41^.

### Limitation

This study is limited in that the participation rate for biochemical validation was low (27.6%, 24 out of 87 at 6 months). Because of the disease and tiredness, many cancer patients who reported quitting showed hesitation to come back for biochemical validation even with financial incentive provided. Other methods of biochemical validation could be explored to increase the participation rate, such as asking subjects to provide samples such as saliva or hair^42^, or visiting subjects’ homes to conduct the test. Another limitation is that the effect size of the intervention in this study was lower than what we expected. Despite the fact that more subjects in the experimental group reported smoking reduction of at least 50% than in the control group at both 6- and 12-month follow-ups, the differences between the two groups were not statistically significant. The results suggest that the relationship between the intervention and the outcomes might have been affected by the limited sample size. It is quite possible that with a larger sample that difference would be statistically significant.

### Implications for Future Practice and Research

The research addressed an important health issue, i.e. to help cancer patients quit smoking, which can lower the risk of disease advancement, minimize adverse treatment-related effects and improve the prognosis and quality of life of patient. The research is original and it helps clarify the effectiveness of a brief smoking cessation intervention to help cancer patients quit. Moreover, this study demonstrated the feasibility of implementing smoking cessation intervention in outpatient clinics. The outcomes of this research help inform future researchers and policy making on smoking cessation for cancer patients and thus have important implications for clinical practice and health significance.

## Conclusion

In conclusion, our smoking cessation intervention based on a risk communication approach was not effective in helping cancer patients to quit smoking. Adding digital reminders could be a future perspective to improve the intervention. In addition, our intervention could be considered as an add-on intervention to existing smoking cessation services to increase the risk perception associated with tobacco use among cancer patients.
